# Multilocus Sequence Typing for Interpreting Blood Isolates of *Staphylococcus epidermidis*

**DOI:** 10.1155/2014/787458

**Published:** 2014-03-02

**Authors:** Prannda Sharma, Ashley E. Satorius, Marika R. Raff, Adriana Rivera, Duane W. Newton, John G. Younger

**Affiliations:** ^1^The Biointerfaces Institute and Department of Emergency Medicine, University of Michigan, 26-329N North Campus Research Complex, 2800 Plymouth Road, Ann Arbor, MI 48109, USA; ^2^Department of Pathology, University of Michigan, 2F461 UH, 1500 E. Medical Center Drive, Ann Arbor, MI 48109, USA

## Abstract

*Staphylococcus epidermidis* is an important cause of nosocomial infection and bacteremia. It is also a common contaminant of blood cultures and, as a result, there is frequently uncertainty as to its diagnostic significance when recovered in the clinical laboratory. One molecular strategy that might be of value in clarifying the interpretation of *S. epidermidis* identified in blood culture is multilocus sequence typing. Here, we examined 100 isolates of this species (50 blood isolates representing true bacteremia, 25 likely contaminant isolates, and 25 skin isolates) and the ability of sequence typing to differentiate them. Three machine learning algorithms (classification regression tree, support vector machine, and nearest neighbor) were employed. Genetic variability was substantial between isolates, with 44 sequence types found in 100 isolates. Sequence types 2 and 5 were most commonly identified. However, among the classification algorithms we employed, none were effective, with CART and SVM both yielding only 73% diagnostic accuracy and nearest neighbor analysis yielding only 53% accuracy. Our data mirror previous studies examining the presence or absence of pathogenic genes in that the overlap between truly significant organisms and contaminants appears to prevent the use of MLST in the clarification of blood cultures recovering *S. epidermidis*.

## 1. Introduction

By nature of their capacity to contaminate and proliferate on the surfaces of implanted medical devices, coagulase-negative staphylococci (CoNS), most often represented by *Staphylococcus epidermidis*, have been estimated to be responsible for 40% of health care associated bloodstream infections [1]. This organism's rise to prominence has paralleled the increased use of short-term intravascular catheters and long-term implantable devices [2–5]. As CoNS are also the most frequent cause of false positive blood cultures, the interpretation of recovering of these organisms from a blood culture is often clinically difficult [6, 7]. Contaminated blood cultures can precipitate unnecessary hospitalization and antibiotic treatment, and the costs associated with follow-up diagnostic testing and therapy of misleading blood cultures are substantial [8]. Better methods of distinguishing recovered isolates likely to represent pathologic bacteremia from isolates inadvertently recovered during the sampling process would be valued. Unfortunately to date, efforts based on determining the presence or absence of recognized pathogenic genes have been unsuccessful [9, 10].

Here, we examine the potential utility of multilocus sequence typing (MLST) in differentiating invasive from commensal strains of *S. epidermidis*, seeking to determine if this epidemiologically useful sequencing strategy might have value in clinical decision-making around the recovery of *S. epidermidis* recovered during the culturing of blood for diagnostic purposes [11].

## 2. Materials and Methods

### 2.1. Source of *S. epidermidis* Strains

Skin isolates were taken from 25 healthy volunteers via thumbprints made into mannitol salt agar plates [12]. All blood isolates were gathered from the University of Michigan Health System Clinical Microbiology Laboratory using the BacT/Alert/FAN system and were identified using Vitek 2 (bioMérieux, Durham, NC) as we have noted previously [6]. The blood cultures were collected within 48 hrs and found to be identical in regard to their antibiotic susceptibility and colony morphology. Hence, the Coagulase-negative staphylococcus isolates were considered to be clinically significant. For purposes of clarity in this report, we regard these isolates as *true positives.* Fifty isolates meeting these criteria were analyzed. Twenty-five additional isolates were identified as *likely contaminants* as they were recovered from only one set of at least two sets of cultures collected over 48 hours.

### 2.2. Genetic Characterization of Isolates

All isolates were confirmed as *S. epidermidis* by sequencing the 16s-23s intergenic spacer region [13]. For MLST, 7 genes (*arcC, aroE, gtr, mutS, pyr, tpiA, and yqiL*) were partially sequenced to determine their type via the online utility at http://sepidermidis.mlst.net/ [14]. Genomic DNA was isolated from the overnight cultures, grown at 37°C, 250 rpm using Wizard gDNA purification kit (Promega number A1120). Later, seven MLST target genes were amplified in 50 uL sample volume containing 100 ng of the gDNA, 0.5 uM forward and reverse primers, 0.2 mM dNTPs (Invitrogen number 18427-013), 2 U Taq polymerase (Invitrogen number 11508-017), 1.5 mM MgCl_2_ and 1X PCR reaction buffer. The PCR products were purified using the Wizard SV Gel and PCR clean up kit (Promega number A9281) then sequenced by the DNA sequencing core at the University of Michigan using an Applied Biosystem 3730XL. Sequence results were analyzed using SEQUENCHER software 4.10.1.

### 2.3. Statistical Methods

Standard odds ratio analyses were used to establish the strengths of association between STs and source of isolate recovery. However, as MLST provides a “7-dimensional” measurement of each strain, multivariate analytical approaches were necessary to evaluate its performance as a diagnostic technology. To develop classification schemes that could be applied in practice to categorize an unknown isolate as representing true bloodstream infection or a likely contaminant, we employed three machine learning algorithms capable of considering all 7 genotypes simultaneously: support vector machine (SVM, that classifies unknown isolates by comparing their MLST alleles relative to an algorithmically generated boundary that best segregates isolates of known class—e.g., pathogen or likely contaminant—on the basis of their alleles); classification and regression tree analysis (CART, which seeks to develop a decision tree against which each allele of each ST could be considered sequentially to predict a strain's identity); and a naïve nearest-neighbor classifier, which assigns an unknown isolate to a group by giving it the same membership as a known strain which it most closely resembles [15–17]. All analyses were carried out in the statistical programming environment R [16].

## 3. Results

**Figure 1:**
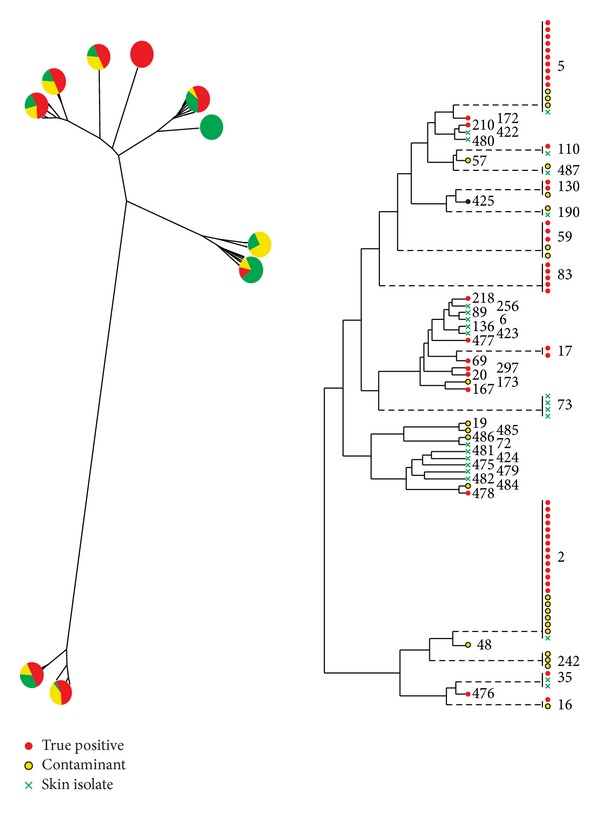
*Staphylococcus epidermidis* dendrogram of relatedness of 50 true positive isolates (red), 25 likely contaminant isolates (yellow) and 25 isolates from the skin of healthy volunteers (green) using multilocus sequence typing. At left, associated pair charts show distribution of isolate source in various arms. At right, numerical values represent the sequence type derived from sequencing of 7 genes of *S. epidermidis*. While in general the horizontal distance between strains depicts their genetic dissimilarity, dashed lines reflect sequence types that have been horizontally shifted to the right to preserve readability of the figure. MLST revealed significant variability in sequence type in this prospectively gathered sample and large overlap in sequence type among strains of different provenance.

Using MLST, we studied 50 true positive blood isolates, 25 likely contaminants, and 25 skin isolates of *S. epidermidis.* Genetic variation was significant, with 44 unique STs identified in collection (Figure 1, with sequence types tabulated in the electronic Supplementary Material available online at http://dx.doi.org/10.1155/2014/787458). Nevertheless, 35 of 100 isolates were either ST2 (21 isolates) or ST5 (14 isolates). ST2 was more likely to be recovered from the blood as either a true pathogen or likely contaminant compared to being recovered from healthy skin (OR 9.3, 95% CI 1.2–75.7). ST5 showed a similar trend with statistically insignificant confidence bounds (6.0, 95% CI 0.7–49.9). Neither sequence type was more likely among true pathogens compared to likely contaminants (ST2, OR 1.2, 95% CI 0.4–3.7; ST5, OR 1.8, 95% CI 0.4–7.4).

We employed three machine learning approaches to examine whether MLST might be useful in the interpretation of blood cultures recovering *S. epidermidis*—classification and regression tree (CART), support vector machine (SVM), and naïve nearest neighbor classification. All three attempt to assign group membership (in this case, “true positives”, those likely representing blood culture contamination and those recovered from the skin of healthy volunteers) to an unknown strain based on the group membership of other isolates with similar genetic background. Given the large heterogeneity between isolates and the frequent cases of particular MLST types appearing only once in the sample, all three classification tools performed poorly. Diagnostic accuracy was 73% for CART, 73% for SVM, and only 53% for nearest neighbor classification. For all three techniques, accuracy was lost primarily because of misclassification of contaminant or skin isolates as significant.

## 4. Discussion

In the current report we describe the use of MLST for distinguishing various isolates of *S. epidermidis*, a task of considerable clinical importance. MLST is a powerful tool for epidemiological and evolutionary studies of bacteria that provides much greater between-strain resolution than traditional antigenic methods such as serotyping, while being more practical and widely available than full-genome sequencing. We were curious if this approach might be used to develop a tool by which blood isolates of *S. epidermidis* might be more readily classified as reflecting true bacteremia as opposed to culture contamination. This was not the case. The amount of genetic heterogeneity among isolates was substantial, with many sequence types appearing only once in the population, making the interpretation of any given sequence type unreliable as a diagnostic test.

While ST2 and 5 were recovered much more commonly among true positive and likely contaminant isolates compared to healthy skin isolates, neither was differentiating in regard to isolate significance or insignificance. However, the rarity of these sequence types among the skin isolates of healthy volunteers is consistent with them possessing some fitness advantage among people ill enough to undergo blood culture. It has been shown in the context of a bone marrow transplantation unit that patient skin flora is dissimilar, on the basis of pathogenic gene frequency, from *S. epidermidis* recovered outside of the health care environment [9]. Our analysis is not able to address whether that association is a reflection of relative pathogenicity of ST2 and 5 or an indication of the prevalence of these isolates in health care environments.

It is not evident to what degree molecular markers alone or in combination will find usefulness in discriminating nuisance isolates from truly pathogenic isolates of CoNS. No study (including the current report) to our knowledge has identified a single marker robust enough to be of clinical value. A panel of genotypic, phenotypic, or perhaps mixed characterization is imaginable, but our work casts some doubt on this approach. At the MLST level, *S. epidermidis* was found to be highly diverse in even a small prospectively gathered sample. Larger samples would be expected to only reveal greater between-isolate variability. Higher resolution genetic approaches (including perhaps whole genome sequencing of many clinical isolates) hold the promise that some collection of markers might be identified. However, the best evidence to data suggests that such diagnostic utility will not be found in the presence of recognized pathogenesis-related genes nor in the housekeeping genes used for MLST typing.

The primary limitation of this work is the relatively small sample considered. Studying only 100 isolates surely prevents us from seeing more subtle patterns in the genotypic data. However, given the wide variety of sequent types recovered in such a small collection, we believe that our primary conclusion—that MLST is unlikely to be of stand-alone value in assessing the significance of a clinically ambiguous blood isolate of *S. epidermidis*—is robust.

## Supplementary Material

A supplemental table of all of our MLST data.
